# Risk factors and predictive nomograms for early death of patients with pancreatic cancer liver metastasis: A large cohort study based on the SEER database and Chinese population

**DOI:** 10.3389/fonc.2022.998445

**Published:** 2022-09-23

**Authors:** Haidong Zhang, Hui Dong, Zheng Pan, Xuanlong Du, Shiwei Liu, Wenjing Xu, Yewei Zhang

**Affiliations:** ^1^ School of Medicine, Southeast University, Nanjing, China; ^2^ Hepatopancreatobiliary Center, Zhongda Hospital, Southeast University, Nanjing, China; ^3^ Hepatopancreatobiliary Center, The Second Affiliated Hospital of Nanjing Medical University, Nanjing, China

**Keywords:** pancreatic cancer, liver metastasis, SEER database, early death, nomogram

## Abstract

**Background:**

The liver is the most common organ for distant metastasis of pancreatic cancer, and patients with pancreatic cancer liver metastases (PCLM) often die in a short period of time. As such, the establishment of an effective nomogram to predict the probability of early death (survival time ≤3 months) in PCLM patients is of considerable significance.

**Methods:**

Patients diagnosed with PCLM in the Surveillance, Epidemiology, and End Result (SEER) database between 2010 and 2015 were included for model construction and internal validation. A data set was obtained from the Chinese population for external validation. Risk factors that contributed to all-cause and cancer-specific early death were determined by means of univariable and multivariable logistic regression. The accuracy of the nomogram was verified by means of receiver operating characteristic (ROC) curves, and the true consistency of the model was assessed by calibration curves. The clinical applicability of the model was evaluated by means of decision curve analysis (DCA).

**Results:**

A total of 12,955 patients were included in the present study, of whom 7,219 (55.7%) experienced early death and 6,973 (53.8%) patients died of PCLM. Through multivariable logistic regression analysis, 11 risk factors associated with all-cause early death and 12 risk factors associated with cancer-specific early death were identified. The area under the curves (AUCs) for all-cause and cancer-specific early death were 0.806 (95% CI: 0.785- 0.827) and 0.808 (95% CI: 0.787- 0.829), respectively. Internal validation showed that the C-indexes of all-cause and cancer-specific early death after bootstrapping (5,000 re-samplings) were 0.805 (95% CI: 0.784-0.826) and 0.807 (95% CI: 0.786-0.828), respectively. As revealed by the calibration curves, the constructed nomograms exhibited good consistency. The decision curve analysis (DCA) indicated the nomograms had significant clinical applicability.

**Conclusion:**

In the present study, reliable nomograms were developed for predicting the early death probability in patients with PCLM. Such tools can help clinicians identify high-risk patients and develop individualized treatment plans as early as possible.

## Introduction

Pancreatic cancer (PCa) is a kind of highly aggressive malignant tumor. The incidence of pancreatic cancer has recently exhibited an upward trend around the world, and an epidemiological survey revealed that PCa ranked fourth as the leading cause of cancer-related death in 2020 (1). Despite the diagnosis and treatment technology of PCa having significantly improved in the past few decades, the five-year survival rate is less than 8% (2). In a study that included 121,713 patients, the results showed that the median survival time for PCa was only 4.4 months, indicating that PCa has a considerably poor prognosis (3). Additionally, once pancreatic cancer metastasizes, the prognosis is disastrous. AJ et al. reported that the 5-year overall survival rate of PCa patients with distant metastasis was less than 3% (2).

As previously reported, the probabilities of peritoneal, liver, lung, bone, and brain metastases from pancreatic cancer were found to be 49.9%, 45.1%, 11.4%, 3.8%, 0.4%, respectively, which indicates that the liver is the most common organ for distant metastasis of PCa (4). Additionally, studies have shown that the prognosis of pancreatic cancer liver metastasis (PCLM) is worse than other distant metastases (5). Thus, the treatment strategy and prognosis of PCa largely depend on whether the patient has liver metastasis.

At present, surgery remains the only available treatment for pancreatic cancer. However, due to the insidious early symptoms and highly aggressive properties of PCa, most patients are diagnosed with PCa when their disease has metastasized. Therefore, most patients have already lost the opportunity for surgery at the time of diagnosis (6). Li et al. reported that only 15%-20% of the patients were eligible for radical surgery for pancreatic cancer at the time of diagnosis (7). However, whether patients with advanced pancreatic cancer can benefit from surgery remains controversial. Dünschede F et al. (8) found that the median survival for patients who underwent synchronous resection of PCa and liver metastasis was not higher than those who only received chemotherapy (8 months vs. 11 months), but Tachezy M et al. (9) conducted a study based on 69 patients with PCLM, which showed that the combined resection of primary PCa and liver metastases could significantly improve the overall survival of patients (14.5 months vs. 7.5 months). In the present study, we will further explore whether patients with PCLM can benefit from surgery.

Currently, chemotherapy is the first-line treatment option for metastatic pancreatic cancer. Conroy T et al. (10) demonstrated that single-agent FOLFIRINOX can prolong the median life expectancy by over 4 months compared with gemcitabine (from 6.8 months to 11.1 months); however, FOLFIRINOX is less safe than gemcitabine. In parallel, phase III trials have shown that nab-paclitaxel combined with gemcitabine can significantly improve the median overall survival of patients compared with gemcitabine alone (8.5 months vs. 6.7 months) (11–13). Despite such improvement, the combined use of drugs had more side effects than the use of a single drug, especially for patients with abnormal liver function, and thus, the combined use of drugs was not recommended. Additionally, studies have shown that even with first-line chemotherapy, the objective response rate is only 50% (14, 15). It can be seen that chemotherapy is effective in improving advanced pancreatic cancer, but the effect is not significant. Therefore, for PCLM patients, there is still no standard treatment regimen and there is no predictive model to accurately predict the chemotherapy effect in patients with PCLM.

Presently, although significant progress has been made in research on the molecular mechanism of PCLM (16, 17), such research can only facilitate understanding of the microscopic pathway of PCLM, and cannot be translated into clinical application. In clinical practice, such research cannot be applied to objectively and accurately assess the prognosis of patients. As such, there is an urgent need for a simple and easy-to-use model for accurate assessment of the prognosis of PCLM patients.

At present, there is a scarcity of research on nomograms for predicting the early death in PCLM and little is known about risk factors for early death in PCLM. In the present study, based on the SEER database and Chinese population, the risk factors for early death in PCLM patients were explored and nomograms were constructed. Such tools are not only beneficial for clinicians in identifying high-risk patients, but also in formulating individualized treatment plans in a timely manner, thereby prolonging the life expectancy, improving the patient’s quality of life, and reducing the economic burden on society and family.

## Materials and methods

### Ethics statement

The present authors received authorization from the National Cancer Institute (USA) (http://seer.cancer.gov) to access the research data in cancer patients (reference number: 17461-Nov2020). The data from SEER database does not require informed consent from patients, and cancer is a reportable disease in every state in the United States. The present study conforms to the 1964 Helsinki Declaration and subsequent amendments or similar ethical standards. In addition, the present study was approved by the ethics committees of Zhongda Hospital Affiliated to Southeast University (approval number: 2022ZDSYLL213-P01).

### Patients

**Figure 1:**
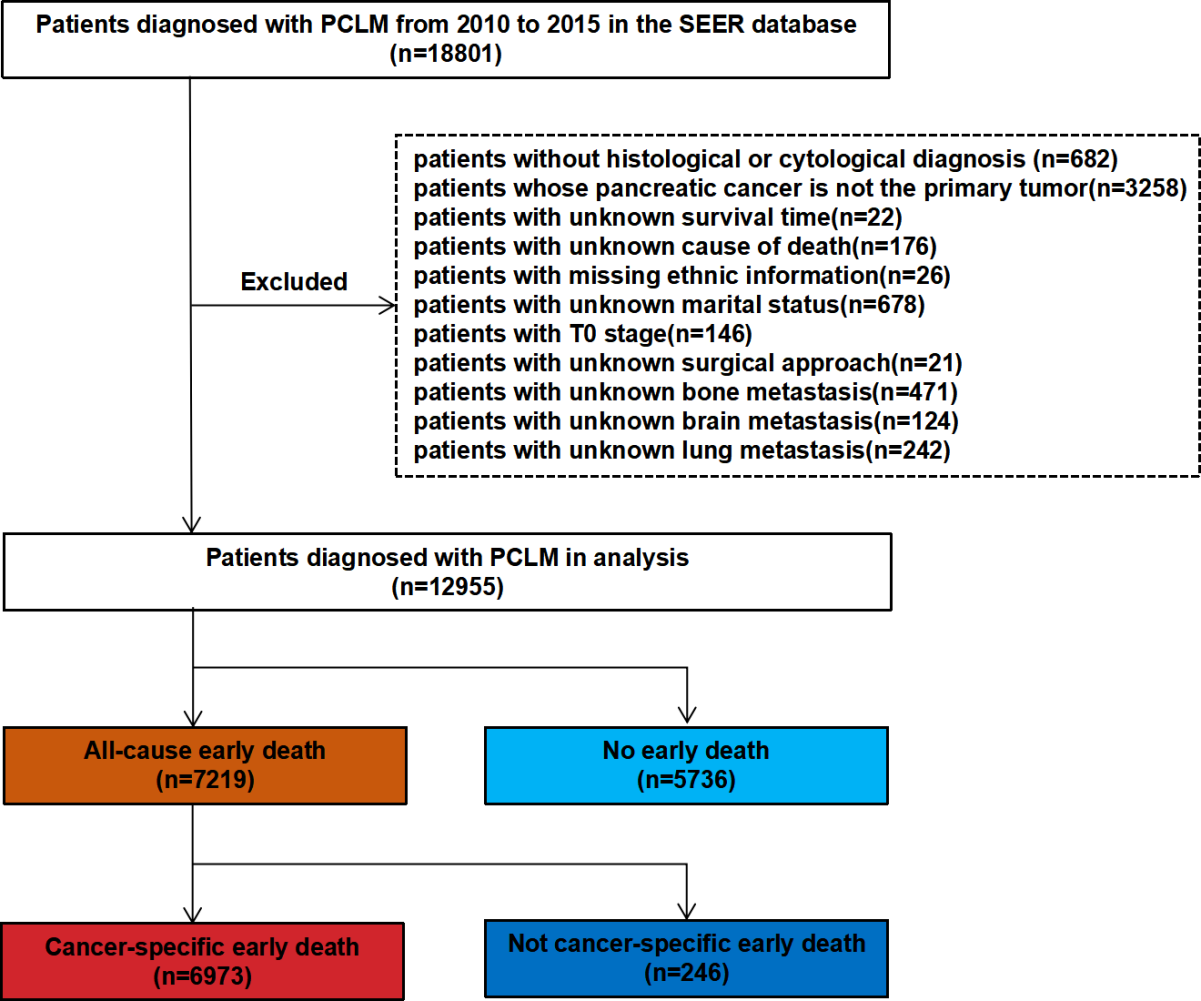
The flowchart of patient selection from SEER database.

In the present study, SEER∗Stat (version 8.3.9.2) was used to extract clinical information of patients with PCLM between 2010 and 2015. The inclusion criteria were as follows: (1) site code: C25.0; and (2) histological codes: 8140/3, 8255/3, 8480/3, 8481/3, 8500/3 [in light of the International Classification of Tumor Diseases Third Edition (ICD-O-3)]. The exclusion criteria included the following: (1) patients without histological or cytological diagnosis; (2) patients whose pancreatic cancer was not the primary tumor; (3) patients with unknown survival time; (4) patients with unknown cause of death; (5) patients with missing ethnic information; (6) patients with unknown marital status; (7) patients with T0 stage cancer; (8) patients with unknown surgical approach; and (9) patients with unknown bone metastasis, brain metastasis, and lung metastasis. **Figure 1** shows the patient screening flowchart. Considering the malignancy of pancreatic cancer and previous studies, early death is defined as death that occurred within 3 months after initial diagnosis. All-cause early death refers to death of a patient due to various causes (such as hypertension, diabetes mellitus, coronary atherosclerotic heart disease, traffic accident, and others.) within 3 months after the initial diagnosis of pancreatic cancer liver metastasis (PCLM). Cancer-specific early death refers to death that can only be attributed to PCLM and has occurred within 3 months after initial diagnosis (18, 19). The starting point for calculating the survival time was the time point of the first histological or cytological diagnosis of PCLM.

Patients’ baseline information (age, gender, race, marital status), tumor characteristics (tumor location, tumor size, histological grade, N stage, bone metastasis, brain metastasis, liver metastasis, and lung metastasis) and treatment records (surgery, radiotherapy, and chemotherapy information) were collected for analysis. For external validation, 131 PCLM patients from Zhongda Hospital Affiliated to Southeast University were used.

### Statistical analysis

Categorical data were described with numbers and percentages (N, %), and Chi-squared tests were used between subgroups. R software (Version 4.1.2; https://www.R-project.org) was used for all statistical analyses. Two-sided *P*-values <0.05 were considered statistically significant. In the SEER data set, univariable and multivariable logistic regression analyses were used to identify the risk factors for early death in PCLM patients. Subsequently, nomogram models were constructed using the R language “rms” package according to the risk factors determined by means of multivariable logistic regression analysis. To evaluate the performance of the nomograms, the AUCs were plotted using the R language “rms” and “ROCR” packages (20, 21). For calibration, nomogram-predicted probabilities were compared with the actual probabilities by means of bootstrapping (1,000 resamples) (22). Decision curve analysis (DCA) could evaluate the clinical applicability of the nomograms, and the DCA curves were plotted to calculate the clinical net benefit rate (23). Bootstrapping (5,000 re-samplings) was used for internal validation, and then the *C*-indexes between the original data and the verification model were compared to assess the accuracy of the nomograms. Data collected from the Chinese population were used to draw calibration plots and ROC curves to externally verify the cancer-specific early death nomograms.

## Results

### Patient characteristics

A total of 18,801 patients diagnosed with PCLM in the SEER database were included in the present study, and after strict exclusion criteria, 12,955 patients were found to meet the research requirements, of which 7,219 (55.7%) patients had all-cause early deaths and 6,973 (53.8%) patients had cancer-specific early deaths. The majority of early deaths occurred in 65 to 79 years of age (45.2%), males (56.1%) and White (78.2%). The pancreatic head (36.3%) was the most common primary site for early death in patients with PCLM. With the exception of patients with unknown differentiation of histological grade, patients with poorly/undifferentiated (12.1%) were more likely to experience early death than those with well/moderately (7.3%) histological differentiation. The lungs (21.1%) were the second most common organ of distant metastasis and the incidences of bone metastasis and brain metastasis were 7.6% and 0.8%, respectively. In terms of treatment, fewer patients underwent surgery (1.7%) and radiotherapy (4.1%), while more patients received chemotherapy (55.9%). For external validation, 131 patients from Zhongda Hospital Affiliated to Southeast University were included in the present study, of which 72 (55.0%) patients experienced early death. Similarly, most patients (43.1%) who experienced early death were between the age of 65 and 79, about 48.6% were male, and the lungs (26.0%) were the second most common site of distant metastases. **Tables 1** and **2** show the demographic and clinical characteristics of PCLM patients in the SEER data set and the external validation data set, respectively.

**Table 1:** Demographic and tumor characteristics of patients with PCLM in SEER database.

All-cause					Cancer-specific	
Characteristics	Overall	Non early death	Early death	Overall	Non early death	Early death
	12,955	5,736 (44.3%)	7,219 (55.7%)	12,587	5,614 (44.6%)	6,973 (55.4%)
**Age (%)**						
<50	832 (6.4)	528 (9.2)	304 (4.2)	812 (6.5)	514 (9.2)	298 (4.3)
50~64	4,822 (37.2)	2,443 (42.6)	2,379 (33.0)	4,700 (37.3)	2,395 (42.7)	2,305 (33.1)
65~79	5,603 (43.2)	2,338 (40.8)	3,265 (45.2)	5,435 (43.2)	2,291 (40.8)	3,144 (45.1)
>=80	1,698 (13.1)	427 (7.4)	1271 (17.6)	1,640 (13.0)	414 (7.4)	1,226 (17.6)
**Gender (%)**						
Female	5,780 (44.6)	2,612 (45.5)	3,168 (43.9)	5,624 (44.7)	2,565 (45.7)	3,059 (43.9)
Male	7,175 (55.4)	3,124 (54.5)	4,051 (56.1)	6,963 (55.3)	3,049 (54.3)	3,914 (56.1)
**Race (%)**						
White	10,230 (79.0)	4,585 (79.9)	5,645 (78.2)	9,982 (79.3)	4,506 (80.3)	5,476 (78.5)
Black	1,762 (13.6)	729 (12.7)	1,033 (14.3)	1,668 (13.3)	695 (12.4)	973 (14.0)
Others	963 (7.4)	422 (7.4)	541 (7.5)	937 (7.4)	413 (7.4)	524 (7.5)
**Marital status (%)**						
Unmarried	2,117 (16.3)	853 (14.9)	1,264 (17.5)	2,028 (16.1)	823 (14.7)	1,205 (17.3)
Married	7,483 (57.8)	3,669 (64.0)	3,814 (52.8)	7,317 (58.1)	3,610 (64.3)	3,707 (53.2)
Others	3,355 (25.9)	1,214 (21.2)	2,141 (29.7)	3,242 (25.8)	1,181 (21.0)	2,061 (29.6)
**Primary site (%)**						
Head	4,700 (36.3)	2,306 (40.2)	2,394 (33.2)	4,556 (36.2)	2,249 (40.1)	2,307 (33.1)
Body	2,064 (15.9)	1,011 (17.6)	1,053 (14.6)	2,012 (16.0)	994 (17.7)	1,018 (14.6)
Tail	2,774 (21.4)	1,127 (19.6)	1,647 (22.8)	2,699 (21.4)	1,106 (19.7)	1,593 (22.8)
Others	3,417 (26.4)	1,292 (22.5)	2,125 (29.4)	3,320 (26.4)	1,265 (22.5)	2,055 (29.5)
**Tumor size(mm)(%)**						
<50	6,923 (53.4)	3,418 (59.6)	3,505 (48.6)	6,737 (53.5)	3,352 (59.7)	3,385 (48.5)
>=50	3,450 (26.6)	1,418 (24.7)	2,032 (28.1)	3,358 (26.7)	1,389 (24.7)	1,969 (28.2)
Uknown	2,582 (19.9)	900 (15.7)	1,682 (23.3)	2,492 (19.8)	873 (15.6)	1,619 (23.2)
**Histological grade (%)**						
Well/Moderately	1,146 (8.8)	619 (10.8)	527 (7.3)	1,117 (8.9)	605 (10.8)	512 (7.3)
Poorly/Undifferentiated	1,482 (11.4)	607 (10.6)	875 (12.1)	1,450 (11.5)	602 (10.7)	848 (12.2)
Unknown	10,327 (79.7)	4,510 (78.6)	5,817 (80.6)	10,020 (79.6)	4,407 (78.5)	5,613 (80.5)
**AJCC N (%)**						
N0	6,636 (51.2)	3,020 (52.6)	3,616 (50.1)	6,442 (51.2)	2,955 (52.6)	3,487 (50.0)
N1	4,246 (32.8)	1,959 (34.2)	2,287 (31.7)	4,131 (32.8)	1,916 (34.1)	2,215 (31.8)
NX	2,073 (16.0)	757 (13.2)	1,316 (18.2)	2,014 (16.0)	743 (13.2)	1,271 (18.2)
**Bone metastasis (%)**						
No	12,129 (93.6)	5,461 (95.2)	6,668 (92.4)	11,777 (93.6)	5,343 (95.2)	6,434 (92.3)
Yes	826 (6.4)	275 (4.8)	551 (7.6)	810 (6.4)	271 (4.8)	539 (7.7)
**Brain metastasis (%)**						
No	12,884 (99.5)	5,724 (99.8)	7,160 (99.2)	12,520 (99.5)	5,603 (99.8)	6,917 (99.2)
Yes	71 (0.5)	12 (0.2)	59 (0.8)	67 (0.5)	11 (0.2)	56 (0.8)
**Lung metastasis (%)**						
No	10,649 (82.2)	4,955 (86.4)	5,694 (78.9)	10,345 (82.2)	4,845 (86.3)	5,500 (78.9)
Yes	2,306 (17.8)	781 (13.6)	1,525 (21.1)	2,242 (17.8)	769 (13.7)	1,473 (21.1)
**Surgery (%)**						
No	12,736 (98.3)	5,565 (97.0)	7,171 (99.3)	12,374 (98.3)	5,448 (97.0)	6,926 (99.3)
Yes	219 (1.7)	171 (3.0)	48 (0.7)	213 (1.7)	166 (3.0)	47 (0.7)
**Radiotherapy (%)**						
No/Unknown	12,420 (95.9)	5,403 (94.2)	7,017 (97.2)	12,058 (95.8)	5,287 (94.2)	6,771 (97.1)
Yes	535 (4.1)	333 (5.8)	202 (2.8)	529 (4.2)	327 (5.8)	202 (2.9)
**Chemotherapy (%)**						
No/Unknown	5,710 (44.1)	906 (15.8)	4,804 (66.5)	5,524 (43.9)	878 (15.6)	4,646 (66.6)
Yes	7,245 (55.9)	4,830 (84.2)	2,415 (33.5)	7,063 (56.1)	4,736 (84.4)	2,327 (33.4)

**Table 2:** Demographic and tumor characteristics of patients with PCLM in Chinese population.

		Cancer-specific	
Characteristics	Overall	Non early death	Early death
	131	59 (45.0%)	72 (55.0%)
**Age (%)**			
<50	10 (7.6)	6 (10.2)	4 (5.6)
50~64	51 (38.9)	24 (40.7)	27 (37.5)
65~79	52 (39.7)	21 (35.6)	31 (43.1)
>=80	18 (13.7)	8 (13.6)	10 (13.9)
**Gender (%)**			
Female	54 (41.2)	17 (28.8)	37 (51.4)
Male	77 (58.8)	42 (71.2)	35 (48.6)
**Marital status (%)**			
Unmarried	2 (1.5)	0 (0.0)	2 (2.8)
Married	106 (80.9)	53 (89.8)	53 (73.6)
Others	23 (17.6)	6 (10.2)	17 (23.6)
**Primary site (%)**			
Head	60 (45.8)	33 (55.9)	27 (37.5)
Body	18 (13.7)	7 (11.9)	11 (15.3)
Tail	38 (29.0)	14 (23.7)	24 (33.3)
Others	15 (11.5)	5 (8.5)	10 (13.9)
**Tumor size(mm)(%)**			
<50	77 (58.8)	35 (59.3)	42 (58.3)
>=50	49 (37.4)	22 (37.3)	27 (37.5)
Uknown	5 (3.8)	2 (3.4)	3 (4.2)
**Histological grade (%)**			
Well/Moderately	18 (13.7)	10 (16.9)	8 (11.1)
Poorly/Undifferentiated	43 (32.8)	21 (35.6)	22 (30.6)
Unknown	70 (53.4)	28 (47.5)	42 (58.3)
**AJCC N (%)**			
N0	28 (21.4)	16 (27.1)	12 (16.7)
N1	103 (78.6)	43 (72.9)	60 (83.3)
NX	0 (0.0)	0 (0.0)	0 (0.0)
**Bone metastasis (%)**			
No	104 (79.4)	50 (84.7)	54 (75.0)
Yes	27 (20.6)	9 (15.3)	18 (25.0)
**Brain metastasis (%)**			
No	129 (98.5)	58 (98.3)	71 (98.6)
Yes	2 (1.5)	1 (1.7)	1 (1.4)
**Lung metastasis (%)**			
No	97 (74.0)	44 (74.6)	53 (73.6)
Yes	34 (26.0)	15 (25.4)	19 (26.4)
**Surgery (%)**			
No	103 (78.6)	35 (59.3)	68 (94.4)
Yes	28 (21.4)	24 (40.7)	4 (5.6)
**Radiotherapy (%)**			
No/Unknown	101 (77.1)	36 (61.0)	65 (90.3)
Yes	30 (22.9)	23 (39.0)	7 (9.7)
**Chemotherapy (%)**			
No/Unknown	59 (45.0)	17 (28.8)	42 (58.3)
Yes	72 (55.0)	42 (71.2)	30 (41.7)

### Risk factor analysis for early death

Univariable and multivariable logistic regression analyses were used to identify risk factors for early death in PCLM patients. In the analysis of all-cause early death, univariable logistic regression analysis revealed that age, race, marital status, primary site, tumor size, histological grade, bone metastasis, brain metastasis, lung metastasis, surgery, radiotherapy, and chemotherapy were significantly related to all-cause early death. Variables with statistical significance in the univariable logistic analysis were included in multivariable logistic analysis, and the results indicated that age, marital status, primary site, tumor size, histological grade, bone metastasis, brain metastasis, lung metastasis, surgery, radiotherapy, and chemotherapy were risk factors for all-cause early death in PCLM patients. In the cancer-specific early death analysis, univariable logistic regression analysis suggested that age, gender, race, marital status, primary site, tumor size, histological grade, bone metastasis, brain metastasis, lung metastasis, surgery, radiotherapy, and chemotherapy were associated with cancer-specific early death in PCLM patients, and multivariable logistic regression analysis showed that age, gender, marital status, primary site, tumor size, histological grade, bone metastasis, brain metastasis, lung metastasis, surgery, radiotherapy, and chemotherapy were risk factors for cancer-specific early death in patients with PCLM. **Tables 3** and **4** illustrate the results of univariable and multivariable logistic analyses.

**Table 3:** The univariable logistic regression analysis of all-cause and cancer-specific early death in PCLM patients.

Characteristics	All-cause early death			Cancer-specific early death		
	OR	95% CI	P-value	OR	95% CI	P-value
**Age (%)**						
<50	Ref			Ref		
50~64	1.691	1.454-1.971	<0.001	1.660	1.424-1.937	<0.001
65~79	2.425	2.088-2.823	<0.001	2.367	2.034-2.759	<0.001
>=80	5.170	4.328-6.187	<0.001	5.108	4.266-6.128	<0.001
**Gender (%)**						
Female	Ref			Ref		
Male	1.069	0.997-1.146	0.060	1.076	1.003-1.155	0.041
**Race (%)**						
White	Ref			Ref		
Black	1.151	1.039-1.275	0.007	1.152	1.037-1.280	0.008
Others	1.041	0.912-1.190	0.552	1.044	0.913-1.195	0.531
**Marital status (%)**						
Unmarried	Ref			Ref		
Married	0.702	0.636-0.774	<0.001	0.701	0.635-0.775	<0.001
Others	1.190	1.064-1.331	0.002	1.192	1.064-1.336	0.003
**Primary site (%)**						
Head	Ref			Ref		
Body	1.003	0.905-1.113	0.951	0.998	0.899-1.109	0.976
Tail	1.408	1.280-1.548	<0.001	1.404	1.275-1.546	<0.001
Others	1.584	1.448- 1.733	<0.001	1.584	1.446- 1.735	<0.001
**Tumor size(mm)(%)**						
<50	Ref			Ref		
>=50	1.397	1.287-1.518	<0.001	1.404	1.291-1.526	<0.001
Uknown	1.823	1.660-2.002	<0.001	1.836	1.670-2.020	<0.001
**Histological grade (%)**						
Well/Moderately	Ref			Ref		
Poorly/Undifferentiated	1.693	1.449-1.979	<0.001	1.665	1.422-1.949	<0.001
Unknown	1.515	1.340-1.713	<0.001	1.505	1.330-1.704	<0.001
**AJCC N (%)**						
N0	Ref			Ref		
N1	0.975	0.902- 1.053	0.521	0.980	0.906- 1.060	0.608
NX	1.452	1.312-1.608	<0.001	1.450	1.308-1.607	<0.001
**Bone metastasis (%)**						
No	Ref			Ref		
Yes	1.641	1.415-1.907	<0.001	1.652	1.423-1.922	<0.001
**Brain metastasis (%)**						
No	Ref			Ref		
Yes	3.931	2.191- 7.683	<0.001	4.124	2.248-8.313	<0.001
**Lung metastasis (%)**						
No	Ref			Ref		
Yes	1.699	1.547-1.868	<0.001	1.687	1.534-1.857	<0.001
**Surgery (%)**						
No	Ref			Ref		
Yes	0.218	0.156- 0.298	<0.001	0.223	0.159- 0.306	<0.001
**Radiotherapy (%)**						
No/Unknown	Ref			Ref		
Yes	0.467	0.390-0.558	<0.001	0.482	0.403-0.576	<0.001
**Chemotherapy (%)**						
No/Unknown	Ref			Ref		
Yes	0.094	0.086-0.103	<0.001	0.093	0.085-0.101	<0.001

**Table 4:** The multivariable logistic regression analysis of all-cause and cancer-specific early death in PCLM patients.

Characteristics	All-cause early death			Cancer-specific early death		
	OR	95% CI	P-value	OR	95% CI	P-value
**Age (%)**						
<50	Ref			Ref		
50~64	1.457	1.221-1.741	<0.001	1.458	1.219-1.748	<0.001
65~79	1.897	1.590-2.267	<0.001	1.900	1.588-2.278	<0.001
>=80	2.496	2.021-3.088	<0.001	2.575	2.077-3.199	<0.001
**Gender (%)**						
Female	NA	NA	NA	Ref		
Male	NA	NA	NA	1.209	1.108-1.320	<0.001
**Race (%)**						
White	Ref			Ref		
Black	1.044	0.923-1.182	0.492	1.061	0.934-1.205	0.363
Others	0.879	0.749-1.032	0.114	0.912	0.776-1.073	0.266
**Marital status (%)**						
Unmarried	Ref			Ref		
Married	0.822	0.729-0.927	0.001	0.813	0.719-0.919	0.001
Others	1.014	0.884-1.162	0.843	1.059	0.920-1.219	0.422
**Primary site (%)**						
Head	Ref			Ref		
Body	1.025	0.905-1.160	0.699	1.015	0.894-1.151	0.822
Tail	1.431	1.277-1.605	<0.001	1.405	1.251-1.578	<0.001
Others	1.302	1.165- 1.456	<0.001	1.297	1.158-1.454	<0.001
**Tumor size(mm)(%)**						
<50	Ref			Ref		
>=50	1.388	1.257-1.534	<0.001	1.378	1.245-1.525	<0.001
Uknown	1.406	1.251-1.580	<0.001	1.418	1.259-1.597	<0.001
**Histological grade (%)**						
Well/Moderately	Ref			Ref		
Poorly/Undifferentiated	1.753	1.457-2.111	<0.001	1.722	1.427-2.080	<0.001
Unknown	1.380	1.191-1.600	<0.001	1.370	1.180-1.591	<0.001
**Bone metastases (%)**						
No	Ref			Ref		
Yes	1.687	1.409-2.022	<0.001	1.677	1.398-2.014	<0.001
**Brain metastasis (%)**						
No	Ref			Ref		
Yes	3.141	1.581-6.684	0.002	3.482	1.710-7.644	0.001
**Lung metastasis (%)**						
No	Ref			Ref		
Yes	1.551	1.387-1.735	<0.001	1.561	1.394-1.749	<0.001
**Surgery (%)**						
No	Ref			Ref		
Yes	0.239	0.163-0.347	<0.001	0.240	0.162- 0.349	<0.001
**Radiotherapy (%)**						
No/Unknown	Ref			Ref		
Yes	0.482	0.386-0.601	<0.001	0.499	0.399-0.623	<0.001
**Chemotherapy (%)**						
No/Unknown	Ref			Ref		
Yes	0.104	0.095-0.114	<0.001	0.103	0.094-0.112	<0.001

NA, Not Available.

### Nomogram construction

**Figure 2:**
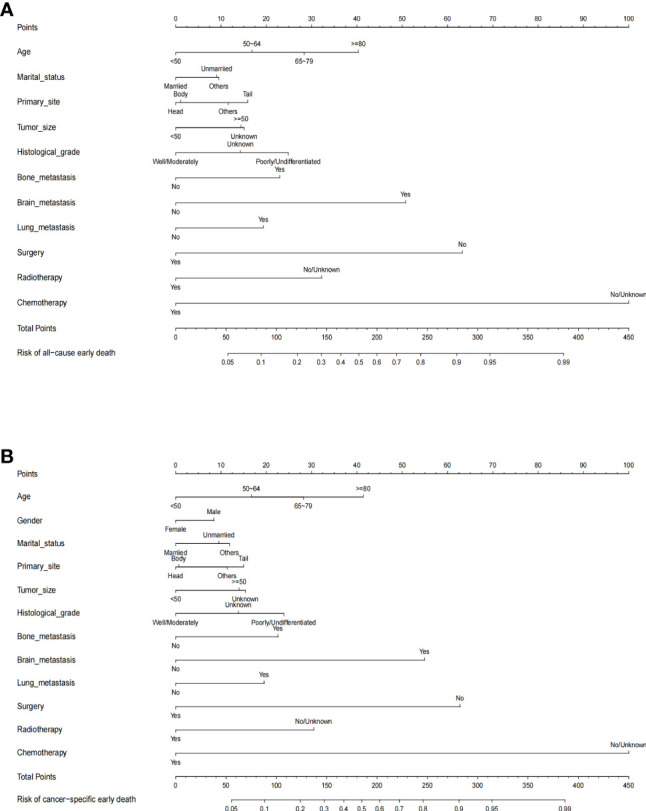
The predictive nomogram for **(A)** all-cause early death and **(B)** cancer-specific early death of pancreatic cancer liver metastasis patients in the SEER database.

The nomograms for predicting the probability of all-cause and cancer-specific early death of PCLM were constructed based on the statistically significant risk factors identified from the multivariable logistic analysis (**Figures 2A, B**). In the nomograms, the total points can be obtained by adding up the scores of each risk factor to predict the probability of early death in PCLM patients. As an example, a 65-year-old, male, married patient with lung metastasis, histologically poorly-differentiated, pancreatic tail cancer, and who had only received chemotherapy, had an approximately 60% chance of early death.

### Performance and validation of nomograms

**Figure 3:**
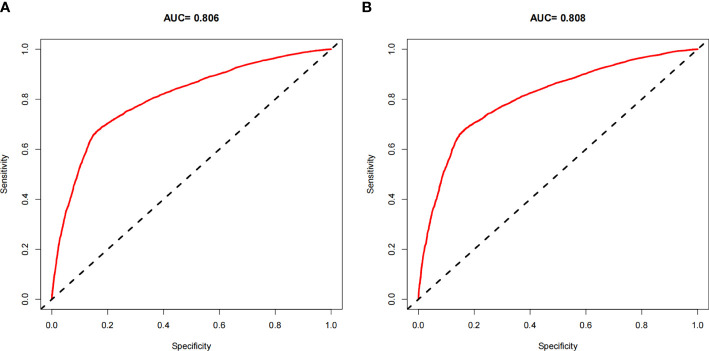
ROC curves for the nomogram. **(A)** The ROC curve for the all-cause early death nomogram in the SEER database; **(B)** The ROC curve for the cancer-specific early death nomogram in the SEER database.

**Figure 4:**
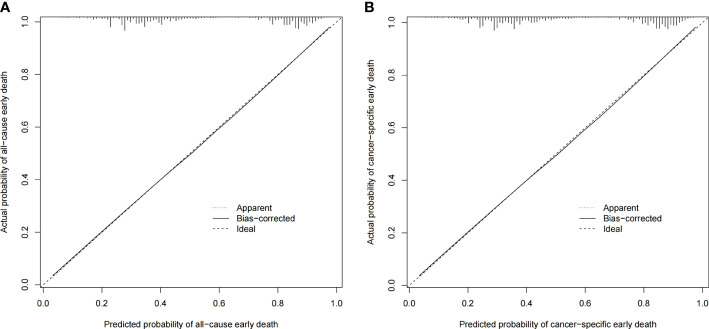
Calibration plots for the nomogram of **(A)** all-cause early death in the SEER database; **(B)** cancer-specific early death in the SEER database.

**Figure 5:**
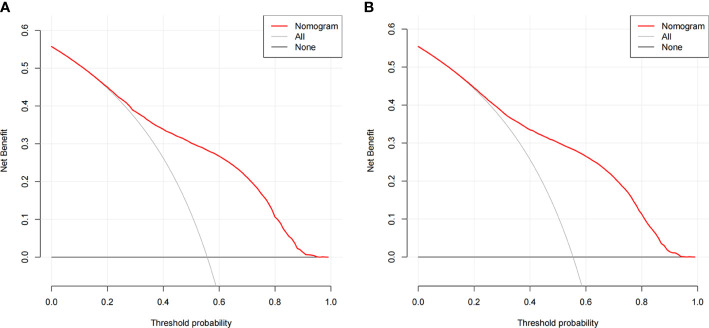
Decision curve analysis **(DCA)** for the nomogram of **(A)** all-cause early death in the SEER database; **(B)** cancer-specific early death in the SEER database.

**Figure 6:**
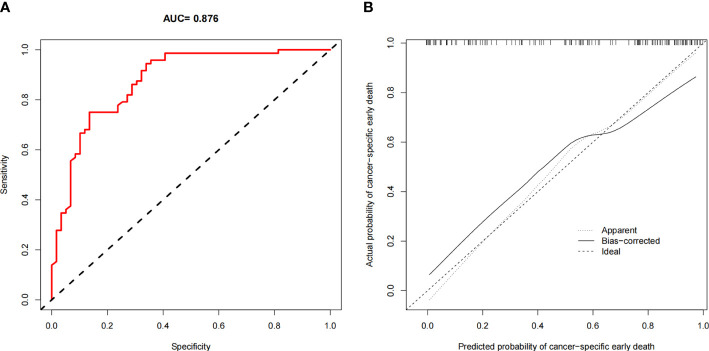
Validation in Chinese population. **(A)** The ROC curve for the nomogram; **(B)** The calibration plots for the nomogram.

The ROC curves of the nomograms for all-cause and cancer-specific early death in PCLM patients are shown in **Figures 3A, B**, revealing that the AUC for all-cause early death was 0.806 (95% CI: 0.785-0.827), and that for tumor-specific early death was 0.808 (95% CI: 0.787-0.829), indicating that the nomogram models had good predictive performance. In the calibration curves, the abscissa represents the predicted probability of early death, and the ordinate represents the actual probability of early death. **Figures 4A, B** show that the predicted curves were always accompanied by the actual curves, indicating that the nomograms have perfect consistency. Bootstrapping was used for internal validation, and the results showed that the C-indexes were 0.805 (95% CI: 0.784-0.826) and 0.807(95% CI: 0.786-0.828) after bootstrapping (5,000 re-samplings) in all-cause and cancer-specific early death analysis, respectively. DCA was used to assess the clinical benefit of the nomograms. **Figures 5A, B** show that in all-cause early death analysis, the nomogram had a favorable threshold probability of 3.0% to 95% and that in cancer-specific early death analysis was 3.0% to 98%. In external validation, only the cancer-specific early death nomogram was validated, since the early deaths in all Chinese patients were caused by PCLM and related factors. The AUC curve **(** **Figure 6A** **)** was 0.876 (95% CI: 0.855-0.897), suggesting that the nomogram had significantly high predictive ability in external validation. Additionally, the calibration curve **(** **Figure 6B** **)** still showed that the nomogram had good consistency.

### Web-based probability calculator

**Figure 7:**
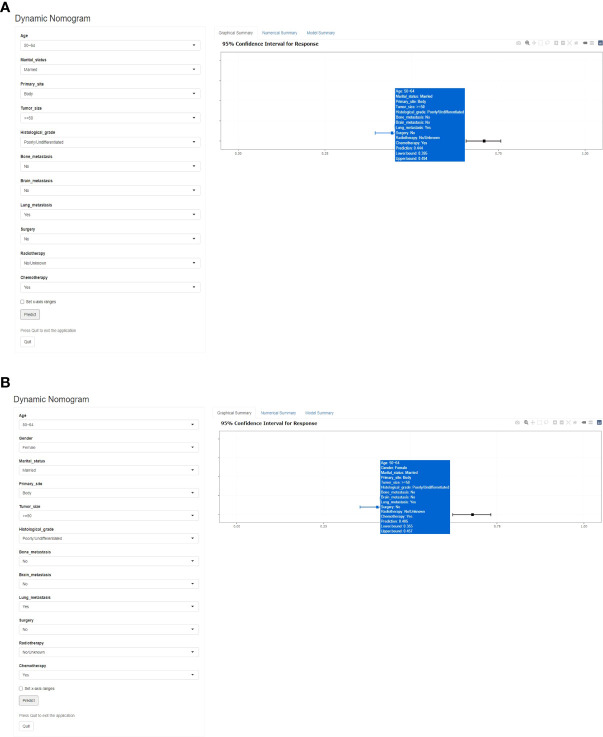
A web-based probability calculator. The graphical summary showed a rough range of **(A)** all-cause and **(B)** cancer-specific early death probability and its 95% confidence interval.

Based on the previous early death predictive nomograms of PCLM, web-based all-cause **(** **Figure 7A** **)** and cancer-specific **(** **Figure 7B** **)** early death probability dynamic calculators were created (https://pclmdynnom.shinyapps.io/DynNomappofall-cause/and https://pclmdynnom.shinyapps.io/DynNomappofcancer-specific/), through which the early death probability could be easily and accurately predicted through inputting the patient’s clinical characteristics.

## Discussion

At present, pancreatic cancer remains a significant global challenge. Although chemotherapy, radiotherapy, and immunotherapy have made considerable progress in the treatment of PCa in recent years, there has been little improvement in the prognosis of PCa (24). Due to the lack of specific symptoms in early PCa, the disease is often at an advanced stage when clinically discovered. The focus of most existing studies has been on the prognosis and survival of early-stage, resectable PCa, however, advanced PCa is more prone to early death. Margaret A et al. reported that the median survival was only 3 months in untreated advanced PCa (25). Further, according to data collected from the SEER database, over 50% of patients with PCLM survive for less than or equal to 3 months. The liver is the most common organ for distant metastasis of pancreatic cancer. Once pancreatic cancer metastasizes to the liver, the liver function will deteriorate sharply, thus early death is more likely to occur in patients with PCLM (4). In the current study, owing to the scarcity of research on PCLM, nomograms for predicting early death in patients with PCLM were developed based on the SEER database and a Chinese cohort was collected to conduct the external validation of the model.

The SEER (Surveillance, Epidemiology, and End Results) database, supported by the NCI (National Cancer Institute), is one of the most large-scale tumor registration databases in America. In the database, 34.6% of the U.S. cancer registry population is recorded (26). Therefore, the results obtained from the SEER database based on multi-center and large sample sizes are more accurate.

The study of early death has been applied to a variety of cancers. For instance, Shen et al. explored the risk factors of early death in patients with brain metastases from lung cancer based on the SEER database and established a nomogram model (18). Song et al. constructed a nomogram for early death of stage IV endometrial carcinoma based on the SEER database, of which the AUC reached 0.877, suggesting that the model had accurate predictive ability (26); however the model was not externally validated. Based on the aforementioned research background, nomogram models for early death of PCLM were established based on the SEER database. Both internal and external validations demonstrated that the established models have good calibration capability.

Although the traditional ROC curve can evaluate the sensitivity and specificity of the model, the clinical practical value of the model cannot be reflected (27). Clinical applicability is a significant indicator for determining whether a patient can benefit from a predictive model; however, there is a scarcity of research in which the method has been applied to evaluate the clinical net benefit rate of predictive models. In the present study, nomogram models of early mortality in PCLM patients were built, and the clinical net benefit rates of the models were calculated. In the all-cause early death model, the threshold probability was 3% to 95%, and that in the cancer-specific early death model was 3% to 98%, which suggests that the present models have great clinical application value. In addition, web-based probability calculators were built, which provide more convenience for clinicians to accurately predict the probability of early death in patients with PCLM, thereby facilitating selection of precise individualized treatment plans as early as possible.

The present study shows that age is an independent influencing factor of early death in PCLM patients, which is consistent with a previous study by Nipp R et al. (28) Additionally, the present study shows that gender is a risk factor for cancer-specific early death, but not for all-cause early death, which may be due to certain confounding factors, such as smoking, drinking, hypertension, and diabetes. However, unfortunately, such unhealthy habits and chronic diseases are not recorded in the SEER database. Further studies have shown that unmarried patients, pancreatic tail tumors, larger tumor size, poor histological differentiation, bone metastases, brain metastases, lung metastases, and patients who have not undergone surgery, radiotherapy and chemotherapy are more prone to early death. Previous studies have shown that such risk factors are indeed associated with prognosis in advanced pancreatic cancer (29, 30), and such factors had an effect on the early death of patients with PCLM in the present study.

Early diagnosis is critical for improving the prognosis of pancreatic cancer. Over the past few years, endoscopic ultrasound-guided fine-needle biopsy (EUS-FNA) has been recognized as the most accurate and advanced diagnostic technique for patients with suspected pancreatic cancer, even for patients with PCLM (31). A prospective study reported that EUS-FNA had a sensitivity of 87.6% and a specificity of 91.2% (32). EUS-FNA can obtain cells or tissue from primary pancreatic cancer and metastases for predictive molecular marker and gene expression analysis. Therefore, if a new nomogram model can be constructed by combining the results of EUS-FNA with the risk factors identified in the present study, it will be more conducive to accurately predicting the prognosis of patients with PCLM and implementing individualized treatment plans.

At present, in addition to chemotherapy, which has been recognized to improve the prognosis of metastatic pancreatic cancer, the immunotherapy for pancreatic cancer has gradually attracted the attention of clinicians and oncologists. However, whether immunotherapy can improve the prognosis of advanced pancreatic cancer is still inconclusive. A retrospective study based on the National Cancer Database (NCDB) revealed that immunotherapy was meaningful in prolonging overall survival (OS) in advanced pancreatic cancer (median OS was 12.2 months in the immunotherapy group versus 5.8 months in the non-immunotherapy group) (33). However, Fan and Ho WJ et al. showed that the effect of immunotherapy in advanced pancreatic cancer was not satisfactory, which may be related to the immunosuppressive tumor microenvironment (TME) (34, 35). Pancreatic immunosuppressive TME comprises myeloid cells, fibroblast, and extracellular matrix (ECM). As such, further understanding of the immunosuppressive tumor microenvironment as well as exploration into the future direction of immunotherapy research is needed, so as to prolong the survival time of pancreatic cancer patients.

Admittedly, there are certain limitations in the present study. First, as a retrospective study, selection bias was unavoidable when implementing exclusion criteria. Second, the SEER database lacks the records of unhealthy habits and the past medical history (such as smoking, drinking, hypertension, diabetes), and certain treatment information is also missing (such as specific chemotherapy regimens). Third, although external validation of the nomograms was performed, the amount of data was insufficient, and multi-center large sample data were still required to ensure the accuracy and extrapolation of the model.

In conclusion, based on the SEER database, nomograms for early death in PCLM patients were constructed, and both internal and external validation indicated that the model had good accuracy. Such tools allow clinicians to identify PCLM patients as early as possible and develop individualized treatment plans, thereby improving the survival outcomes of PCLM patients.

## Data availability statement

Publicly available datasets were analyzed in this study. This data can be found here: (http://seer.cancer.gov). More specific data used during the present study are available from the corresponding author upon reasonable request.

## Ethics statement

All experimental protocols were approved by the National Cancer Institute (USA) to obtain research data on cancer patients (reference number: 17461-Nov2020). Data collected from the Chinese population was approved by the ethics committees of Zhongda Hospital Affiliated to Southeast University (approval number: 2022ZDSYLL213-P01). This study was in line with the 1964 Helsinki Declaration and subsequent amendments or similar ethical standards.

## Author contributions

HZ and YZ contributed to the study design and literature search. HZ, HD, and SL completed the data analysis. ZP, XD, and WX generated and improved the figures and tables. HZ completed the manuscript. YZ proofread the manuscript. All authors contributed to the article and approved the submitted version.

## Fundings

The present study was funded by 1: The National Natural Science Foundation of China. (81872255, 62141109) 2: The Leading-edge Technology Programme of Jiangsu Natural Science Foundation. (BK20212012)

## Acknowledgments

The authors are grateful to all the patients, researchers, and institutions that participated in the SEER database and Chinese population.

## Conflict of interest

The authors declare that the research was conducted in the absence of any commercial or financial relationships that could be construed as a potential conflict of interest.

## Publisher’s note

All claims expressed in this article are solely those of the authors and do not necessarily represent those of their affiliated organizations, or those of the publisher, the editors and the reviewers. Any product that may be evaluated in this article, or claim that may be made by its manufacturer, is not guaranteed or endorsed by the publisher.

## Abbreviations

PCa: pancreatic cancer; PCLM: pancreatic cancer liver metastasis; SEER: Surveillance, Epidemiology, and End Results; C-index: concordance index; ROC: receiver operating characteristic; AUC: area under the curve; DCA: decision curve analysis; NCI: National Cancer Institute; NCDB: National Cancer Database; OS: overall survival; EUS-FNA: endoscopic ultrasound-guided fine-needle biopsy; TME: tumor microenvironment; ECM: extracellular matrix; ICD-O-3: International Classification of Tumor Diseases Third Edition; OR: odds ratio; CI: confidence interval; AJCC: American Joint Commission on Cancer.
